# Hospital and Institutional News

**Published:** 1915-03-06

**Authors:** 


### HOSPITAL AND INSTITUTIONAL NEWS.

### PAYMENT FOR WOUNDED SOLDIERS: OFFICIAL STATEMENT.

In connection with this subject, which has naturally been much exercising the minds of hospital managers during the past few months with increasing force, the British Hospitals Association make an announcement of some interest. The Association have previously issued a circular-letter to the hospitals, and now add the following communication:—"Referring to our previous circular-letter upon this subject, we now have to inform you that an application was made to the War Office, and a reply has been received to the following effect: 'That the general principle adopted as to payment for hospital accommodation for wounded soldiers is that the hospital concerned should take up the question with the general officers of the command or district in which the hospital is situated, with a view to fixing a reasonable rate of payment, having regard to the circumstances of each case. It will be obvious that considerable variation in the rates paid is inevitable in the circumstances, but the Council are of opinion that the system adopted is that best suited to meet the needs of the situation, and has, so far as they know, worked satisfactorily hitherto.' In these circumstances, the Council of the British Hospitals Association recommend the voluntary hospitals who have not already made arrangements for payment, but who desire payment to be made for these patients, to apply to the local military authorities, as suggested by the War Office." On this we may ask if it is indeed so obvious that "considerable variation in the rates paid is inevitable," and should be glad to have the opinions of hospital managers on the desirability and possibility of a more uniform scale?

### BOMBARDMENTS AND ANNUAL REPORTS.

What is probably the first annual report to contain direct personal evidence not merely of the war, but of the German raids on our shores, is that of the Scarborough Hospital and Dispensary. This historical document records that, as the result of the bombardment on December 16 the hospital buildings were struck in two places, and damage to the amount of £63 (covered by a war-risk insurance policy) occurred. Many cases of injury from the firing, it also states, were treated both in the hospital and the out-patients' department. The matron, Miss Richardson, left in October to engage in Army hospital work in France, and Sister Cooper was appointed to undertake the duties until her return. The medical staff was much disorganised during the last months of the year. Dr. Giles volunteered and was accepted for service at the Front. Mr. Cecil Ashby joined the Forces. Dr. Libbey, one of the honorary anæsthetists, was called up for service, and for a considerable time the hospital was without the services of male house surgeons. The board, however, secured the services of Dr. Isabella Little, and adds the now proverbial comment that "the dearth of house surgeons is causing much inconvenience to all hospitals." The board placed twenty beds at the disposal of the military authorities and a similar number at the disposal of the naval authorities. A number of wounded Belgian soldiers have been treated in the wards.

### THE GERMAN HOSPITAL SINCE THE WAR.

Naturally, under the present circumstances, peculiar interest attaches to the first (and, we hope, last) annual meeting of the German Hospital, Dalston, since the outbreak of war. The court was held last week, and a few of the bare facts stated in the report are worth recording, though the hospital's situation has not greatly or outwardly differed much from that of its sister institutions. No sooner was war declared than a special meeting of the committee and medical officers was called, at which the situation was debated, with the result that it was determined to continue the work of the hospital as usual, so long as a sufficient staff could be maintained. A lack of medical officers was immediately felt, and out-patient treatment had to be restricted to serious or urgent cases, while the eastern dispensary was closed down. In-patients, however, have been admitted without interruption; indeed, the demand for beds became so great that a temporary addition of thirty-nine beds has been provided by the honorary treasurer, Baron von Schroder. The resident medical staff was quickly depleted by resignations, but the difficulty consequent on this was obviated through the kindness of the honorary visiting staff, two of whom successively took up their residence in the hospital, while other members also devoted more time to the institution. Eventually two house physicians and one house surgeon were secured, who are still undertaking the work. As regards the war, the committee of the German Hospital placed fifty beds in the hospital and ten in the convalescent home at Hitchin at the disposal of the authorities for the reception of the wounded. The offers were acknowledged with thanks. Among the patients have been sixty-eight prisoners of war, sent to the hospital from the various detention camps and ships. While the completion of the new residential block has set free the rooms in the hospital formerly occupied by the resident staff for a medical library, writing and sewing rooms, etc., the work of building a new mortuary unit has been postponed.

### NORTH EVINGTON INFIRMARY: THE GUARDIANS' DECISION.

The Leicester Board of Guardians have unanimously decided to comply with the suggestion of the War Office to place the North Evington Infirmary at their disposal. The house committee, in introducing the matter, stated that the War Office would bear any extra expense the Guardians might be put to in making fresh arrangements for the patients under the Guardians' care. The Hon. Gerald Walsh, Local Government Board Inspector, stated that the War Office was particularly anxious to acquire the infirmary for the reasons that Leicester was not far from London, that there was an excellent train service, and that there were in Leicester capable surgeons and medical men. The Clerk to the Guardians, Mr. Mansfield, indicated in general outline the arrangements for the care of the 500 or more patients under the Guardians' charge. Some would be accommodated in the old workhouse, where additional accommodation could readily be brought into existence, others at the Guardians' receiving home in Mill Hill Lane, and others at the Cottage Homes at Countesthorpe, where there was ample room for extension. He suggested that an endeavour be made to arrange with the Local Education Authorities for one of the elementary schools to be placed at the Board's disposal for the accommodation of other inmates, and that the Sanitary Committee of the Town Council should take charge of the consumptives. The approval of the Guardians was readily given to the acquisition of the infirmary and to the suggested arrangements for the accommodation of patients, and a committee was appointed to confer with the Local Sanitary Committee with reference to the care of consumptives.

### THE DISADVANTAGES OF SCHOOLS AS TEMPORARY HOSPITALS.

In our last issue we stated that school buildings were structually inadequate for hospital purposes. It follows, therefore, that to attempt to make them adequate would necessitate a large expenditure of time and money, and even then efficiency would not be secured. An outline of the provision which it would be necessary to make may be gathered from the assumption that the entire drainage system would have to be overhauled and considerably extended; extensive and separate lavatory accommodation provided for doctors, nurses, orderlies, and patients; slop sinks, adequate bathrooms, and other appliances fitted; bedrooms and bathrooms for nurses, orderlies, and staff provided; dining rooms, mess rooms, sick rooms, and off-duty rooms for nurses, orderlies, and staff arranged; heating installations increased and additional power installed; the hot and cold water system considerably extended; incinerator installed; and facilities provided for the isolation of infectious and acutely septic cases; large storage and office accommodation also would be indispensable. We have dealt with these disadvantages somewhat fully to accentuate the advantages of using the 5th Northern General Hospital and the North Evington Infirmary for additional accommodation. Both sites are attractively situated, have fine altitudes, and although very central are not in any way surrounded by other buildings. It is also quite possible that the limit of accommodation has not been reached in those hospitals which have already placed beds at the disposal of the Government. The Mayor of Leicester has been in consultation with those who are in a position to advise upon this matter, and has made suggestions to the War Office with a view to the provision of better accommodation than could possibly be found by using the elementary schools. We do not doubt that these suggestions, which have not been made public, follow very much on the lines we have sketched out. A conference between the various authorities is essential if the best is to be made of the favourable resources of Leicester.

### THE NEW MEDICAL SCHOOL AT CARDIFF.

Full authority, it is understood, has been given to proceed with the building of the new medical school at Cardiff, which means that the Treasury will recognise the work to be carried on there and be prepared from time to time to give substantial grants for the purposes of research. The extent of the Treasury aid is not disclosed, but the project has commended itself not only to the Treasury officials and to the Chancellor of the Exchequer, but to the Inter-Departmental Committee, upon whose report the Treasury has based its conclusions. The plans prepared by Colonel Bruce Vaughan, the architect for the building, have been approved, and the erection of the physiological block, the contract for which has been already signed as between Sir William James Thomas, the donor of £90,000, and the contractors, Messrs. Turner and Co., will be begun immediately.

### REMARKABLE HOSPITAL AND RED CROSS CHAPELS.

In view of the articles on "Remarkable Hospital Chapels," which have lately been appearing in our columns, and to which we invite our readers to contribute further examples, it is interesting to note that the mortuary chapel of the new King George (Red Cross) Hospital, near Waterloo Station, has received an important gift from Queen Alexandra. As President of the British Red Cross Society, she has presented a brass cross and two brass vases for the Holy Table in the mortuary chapel at the institution. The cross is embellished with moonstones and bears the following inscription on the steps of its base: "Jesus Calls. Now Comes Peace. From Alexandra." We have so often insisted upon the need for maintaining the highest standard of reverence for the dead in hospitals, which are the great teachers in this as in many other respects to large sections of the nation, that the present gift to the mortuary chapel of the King George (Red Cross) Hospital should serve as a graceful and practical reminder of what a hospital mortuary chapel should be.

### THE NUISANCE AT SOUTHWARK INFIRMARY.

The Southwark Board of Guardians have at last been informed of a method whereby they can compel the Camberwell Borough Council to abate the nuisance arising from their notorious refuse siding near the Southwark Infirmary at East Dulwich. The Local Government Board, to whom they appealed after months of delay, informed the Guardians they had no powers by which they could compel the Camberwell Council to do their duty as a public health authority, and referred them to the London County Council. Fortunately, last summer did not see a return of the enteritis among the children in the infirmary in the aggravated form of the previous year, though the nuisance from the refuse siding still remained. The London County Council have now informed the Guardians that they were advised that there was no evidence of nuisance at the present time, and they were unable to institute proceedings against the Camberwell Borough Council under section 22 of the Public Health (London) Act, 1891. If a certificate was given to the County Council by two medical officers or by ten inhabitants of the district that within the past six months the premises in question have been a nuisance or injurious or dangerous to the health of any of the inhabitants of the district in accordance with the provisions of section 21 of the Act, the Council can then institute proceedings in respect of the nuisance. The certificate must allege specific instances and dates when the nuisance was observed. Such a simple way out of the difficulty, which has been unduly prolonged, had not been submitted to the Guardians, not even by the officials of the Local Government Board. Everything now depends upon the Guardians having the necessary evidence in the specific form required. The infirmary committee are considering the matter with a view to taking action.

### THE EFFECT OF MARRIAGE ON THE MORTALITY OF WOMEN.

Three life-tables of an entirely new description are included in the recently published Supplement to the Seventy-fifth Annual Report of the Registrar-General of Births, Deaths, and Marriages in England and Wales. They refer to female lives, and the distinguishing feature is that the rates are analysed according to marital condition. These tables show that "in the earlier years of married life the wives suffer from a heavier mortality than do their spinster sisters, but from about age forty-five to fifty-five the position is reversed, and still later in life there is not much difference between the two classes. Throughout life until old age widows suffer from a heavy rate of mortality, but above about age eighty all the three classes—single, married, and widowed—are scarcely distinguishable." These figures would seem to show that a woman deciding to marry may expect to risk her health in the early years of matrimony, but that, if she survives these, she may look forward to a middle age of greater vigour than her unmarried sister. The perils of spinsterhood are found to lurk in middle life, but if the unmarried woman successfully sails these perilous seas, she and the married woman may be equally healthy in the autumn of their days—a conclusion which bears meditating upon. Those under the common illusion of Ecclesiastes and Hamlet, that life should be measured by its excess of pleasures over pains, will, therefore, ask themselves whether they prefer a healthy youth or a healthy middle life. Fortunately women, as a sex, are less likely to side-track themselves in this way. Hedda Gablers exist, but there are fewer Heddas than Hamlets.

### THE OUTSIDE TEACHING OF MIDWIVES.

In April, 1914, the Central Midwives Board adopted the following resolutions: "(1) That it is desirable that the lectures in London and the London district, required to be attended by candidates for the Board's diploma, should be given in convenient centres and by experienced lecturers; (2) that notice be accordingly given to the lecturers in London and the London district not attached to institutions; that the recognition of lecturers in London and the district is under consideration, and that it may not be renewed on its expiry on March 31, 1915." In pursuance of these resolutions the Board has now given notice to the lecturers not connected with institutions that they will no longer be recognised as teachers of pupil midwives. This decision will probably meet with an energetic protest. In many of the Poor-Law infirmaries the number of maternity cases admitted will allow of the training of only three or four pupil midwives annually. In such cases it has been customary for the pupils to go to an outside teacher for their theoretical training, with an obvious advantage as regards economy of labour. Hitherto the nurses have had a fairly large choice, and many experienced medical men unconnected with institutions have devoted themselves to the work. Quite apart from the stigma on these men implied by this action of the Board, the field of choice will now be severely and artificially limited. That justification for the Board's action on the score of inefficiency is not obvious is shown by the marked success gained by many of the teachers who are now to be superseded. The Board's powers have not always been exercised wisely in the past, and this last example of interference is one which will not go unchallenged.

### A TESTATOR ON INVESTING LEGACIES.

Very valuable bequests for the benefit of Glasgow hospitals and other charities arise under the will of Miss M. S. Schaw of that city. She bequeathed, free of legacy duty, to the Glasgow Royal Infirmary and the Glasgow Western Infirmary £5,000 each. But Miss Schaw added the condition that, while the legacies shall be paid over respectively to the treasurers or other officials for the time being, the capital of these sums shall not be expended, but shall be held and invested, and the interest or the annual proceeds only applied towards the objects and support of the respective institutions. She also bequeathed £2,000 to the Royal Hospital for Sick Children, Glasgow, to endow a ward to be called the Marjory Shanks Schaw Ward, and in case of there being no ward available to endow a bed or beds in the hospital to be named after herself in the same way. The emphasis which Miss Schaw laid upon the value of investing legacies is noteworthy, and, though these restrictions are not popular among legatees, they certainly have their value.

### CASUALTIES IN THE FRENCH MEDICAL SERVICE.

Casualties in the French Army Medical Service have been very heavy, much heavier, in fact, than in the German Medical Service. According to the Paris correspondent to the official organ of the American Medical Association, there were at the end of the year 14,000 military doctors in France, of whom 6,500 were with the Armies. The number of killed up to that date was 93; 260 were wounded and 440 were missing; and 507 were sick. Thus, altogether, one-fifth of the total number with the Armies were killed, wounded, missing, or incapacitated. The bravery and distinguished conduct of members of the French Medical Service have been freely recognised by the authorities, 155 names having been mentioned in dispatches, while 63 Army doctors have been nominated Chevaliers of the Legion of Honour and 15 as officers of the same Order. Eleven auxiliary physicians have received the military medal. Dr. Dercle, one of those who have been decorated with the Cross of a Chevalier of the Legion of Honour, bears the marks of ninety-seven wounds.

### THE LIVERPOOL PORTABLE HOSPITAL.

Allusion has been made to the Liverpool Merchants' Mobile Hospital, now in course of erection; and as a portable institution all details concerning it are of interest. A specimen section was exhibited last week, consisting of four blocks—the administrative block, the theatre block, and two wards. When complete the hospital, it is understood, will extend over a site of four acres, and will include eight wards, on the pavilion system, for the accommodation of 220 patients. In addition there will be, of course, an administrative block and a theatre unit, while the various departments of the institution will be connected together by means of covered corridors. Each ward is furnished with a shutter, to enable the beds to be wheeled out in fine weather. The floor and the walls are of spruce, painted green on the outside, the former having a damp-proof lining, and the various sections are connected by water-tight joints, formed with grooved surfaces and then bolted together. The inside walls are lined with felting to afford protection from the weather, and to maintain an even temperature. The roofs are made water-tight with a covering of "rubberoid." Heating is to be secured by stoves, and lighting by oil lamps; while the kitchens and laundry are to be supplied with steam. The cost of the building is £13,000, and the equipment, other than surgical appliances, has been supplied in the main by Messrs. G. H. Lee and Co., of Liverpool.

### THE SELECTION OF SANATORIA CASES: A NEW WHOLE-TIME POST.

Owing to the additional work which has fallen upon the Public Health Department of the London County Council in connection with the scheme for the treatment of tuberculous persons it has been found necessary to detach one of the assistant medical officers, Dr. F. N. K. Menzies, from his regular work and devote practically the whole of his time to this new sphere. The new work which falls upon the department is considerable in extent and important in character. It involves the inspection of sanatoria and hospitals with which the County Council is in negotiation for the provision of suitable accommodation, the interviewing of secretaries and committees of the providing bodies and of medical officers with regard to the dispensary provision, and consideration of cases nominated for treatment under the scheme and the selection of cases suitable for sanatorium treatment. Up to the present 300 of such cases have been dealt with, and it is anticipated that this number will be considerably increased in the near future when the dispensary schemes of the various borough councils are in full operation. It is now proposed to detach Dr. Menzies entirely for this work, and to appoint a successor to carry on the duties formerly allotted to him as the divisional medical officer in charge of the East End Division of London.

### THE SITE OF ST. KATHARINE'S.

The Local Government Committee of the London County Council recently requested that body to record its opinion that the claims of the district contiguous to, and in the immediate neighbourhood of, the original site of St. Katharine's Hospital foundation should be adequately recognised in the new scheme. The question arose, as already reported in The Hospital, out of the fact that premises at Poplar had been taken temporarily as a college for the provision of health visitors. The Council, however, referred back the recommendation. The Stepney Borough Council has since urged that all possible steps should be taken to see that the college should be established at Stepney, and it has addressed a petition on the subject to Queen Alexandra, the patron. The Local Government Committee now intimate that there is some prospect of the matter being reconsidered, and they are to report any developments which may take place.

### ANOTHER SCHEME HELD UP BY THE WAR.

Partly owing to the new work with which they have been somewhat overwhelmed, and partly to the difficulty of raising or maintaining income, enlargement schemes in different parts of the country have often had to be curtailed or given up. Thus special interest attached to what Mr. Frederick W. Hunt would be likely to say when he took the chair last week at the annual meeting of Queen Charlotte's Lying-in Hospital. The report stated that a special feature of the work had been the treatment of the wives of our sailors and soldiers. From the outbreak of war until the end of 1914, 160 wives of sailors and soldiers have been granted admission without the usual subscriber's letter and free of charge. Similarly, 218 others have been attended in their own homes. In addition a certain number of Belgian refugee women have been admitted. Great difficulty, it seems, has been experienced in obtaining funds for maintenance. No legacies have been received. Mr. Hunt, however, referred to the scheme of enlargement, which includes an out-patient department, new dining rooms, lecture room, and a home for district midwives and nurses. All these it was hoped to have been able to begin some months ago. War broke out, however, just as the committee were on the point of making a contract, and it was decided to postpone the matter. He trusted that before long they would be able to begin the extension, for the work of the hospital had been carried on for months under very cramped and difficult conditions. The scheme, he added, had been passed by King Edward's Hospital Fund, which had made a grant of £1,000 towards the cost. Over £4,000 had been received from other sources. But there still remains over £10,000 to be raised to pay for the cost of the site and buildings. The Ladies' Association—which, by the way, maintains a bed in the hospital by a subscription of £50—continues to grow, and should, indeed, in such an institution as Queen Charlotte's play an even greater part than it has yet done.

### THE HOSPITAL FOR INDIANS AT BROCKENHURST.

The public hears less than it did about the Indians in the New Forest, though the early days of their arrival there produced a crop of visitors, descriptions, and photographs. Yet a permanent feature, so far as the war is concerned, was added by the opening six weeks ago, on January 20, of the Lady Hardinge Hospital for wounded Indian soldiers in Brockenhurst Park. It contains over three hundred patients in buildings—that is, carefully designed huts—erected by the War Office. The hospital, however, is supported by the Indian Soldiers' Fund, formed by the committee of ladies of the Order of St. John of Jerusalem in England. The site was given by Lady Morant, and the equipment has been provided by the Order of St. John of Jerusalem, which gave £10,000 for the purpose. The hospital is in charge of Lieutenant-Colonel Perry, C.I.E., who was formerly Principal of the Lahore Medical College, under whom is Lieutenant-Colonel Meyer, formerly Professor at the Grant Medical College of Bombay. The staff consists of eight other officers of the Indian Medical Service, each one of whom has a record of distinction. The matron is Miss McCall Anderson, who has sixteen trained nurses under her, all of whom speak Hindustani. They are not responsible for any nursing, but their duties consist in superintending the domestic and sanitary work of the institution. There are also thirty English orderlies, a native staff of forty persons—"havildars, cooks, *bhistis* "—all men from whose hands, it is stated, no man of any caste need hesitate to take a drink. Sir John Hewett, chairman of the Indian Sub-Committee of the Order of St. John of Jerusalem, was among the group of visitors which inspected the institution last Saturday.

### WOMEN DOCTORS AND THE WOUNDED.

Through the war, women have added in a very remarkable way to the laurels we all knew from experience that they would win in the field of nursing. At the recent meeting at Sunderland House on behalf of the extension of the London School of Medicine for Women, Surgeon-General Sir Alfred Keogh highly praised the work done by the women doctors in their hospitals in Paris and Wimereux. He had been so impressed, he said, by the value of their services that he had invited Dr. Flora Murray and Dr. Garrett Anderson to come to England and take charge of a hospital of 500 beds. Dr. Flora Murray and Dr. Garrett Anderson closed their two hospitals in France in the middle of January. Their institutions at Claridge's in Paris under the French Red Cross, containing 150 beds, and at Wimereux under the War Office, containing 80 beds, have been less needed, and Dr. Murray and Dr. Anderson, having accepted Sir Alfred Keogh's offer, are in London. The new hospital will contain 500 beds and be staffed by women, women orderlies working under sisters. It is hoped to open the hospital about the middle or the end of April on a site not yet decided upon. The German War Office has recently appointed Dr. Elizabeth Reinecke its first woman army doctor, to take charge of a military hospital (*Lazarettärztin*). In Russia women army doctors are allowed at the Front, so long as their numbers do not exceed half that of their male colleagues.

### COTTAGE HOSPITALS AND THE WAR.

While cottage hospitals have less opportunity of appearing among the institutions which have given prominent help during the war to our soldiers, yet it would be a mistake to suppose that they are not doing their share. The case of the Southwold Cottage Hospital may be taken as an instance. While its committee decided not to offer the institution and the services of its staff to the War Office, yet they placed their resources and appliances at the disposal of the emergency hospital opened by the cottage hospital's president, Lord Stradbroke, at Henham Hall. In acknowledging the offer of this assistance the Countess of Stradbroke stated that any expense incurred on this head should be refunded. Several members of the staff have enlisted, among whom is the matron, Miss J. L. Riley, who has taken a post at the new King George (Red Cross) Hospital in London. After the war she will return to Southwold. Owing to the war certain important annual sources of income, including a bazaar and a street collection, have been abandoned. The $x$-ray department has been much used, and thirty-five wounded soldiers from Henham Hall have also been examined. This is no bad record for a cottage hospital, and an account of it may serve the double purpose of consolidating public support to these institutions and of encouraging other cottage hospitals to feel that they, too, have a useful part to play in hospital work connected with the war.

### WINWICK MENTAL HOSPITAL FOR WOUNDED SOLDIERS.

The decision of the Lancashire Asylums Board to convert the Winwick Asylum into a hospital for wounded soldiers has very quickly given rise to a spirited protest. It appears that the War Office has asked various authorities in Lancashire and Cheshire to provide 15,000 beds by the end of March, and the Asylums Board, by their decision just mentioned, will be able to provide accommodation for some 4,000 soldiers. The 2,200 patients now housed at the Winwick institution will be transferred to other mental institutions in the two counties. Speaking at a meeting of the South Manchester Board of Guardians last week, Mr. E. Melia, chairman, said that if the Winwick patients were distributed to the other county mental hospitals, already overcrowded, it would amount to a public scandal. Indeed, hundreds of unfortunate patients, said Mr. Melia, were at present housed in the totally unsuitable environment of their local workhouse. In September of 1914 there were 3,228 mental patients in the thirty Unions of the county, the numbers varying in different institutions, from one at Garstang to 313 at Withington, the South Manchester institution. These patients were not kept or housed as they required to be, and they ought not to be left in the workhouses for one moment longer than was necessary. The point raised by Mr. Melia was that, while they were prepared to do everything possible to meet the needs of the wounded, there were other buildings which ought to be exhausted before the mental hospital accommodation was restricted. In supporting this latter statement, Mrs. Garrett mentioned Eaton Hall, Chatsworth House, and Welbeck Abbey. A hasty statement also fell from Mrs. Garrett to the effect that the way in which wounded men were knocked about in hole-and-corner hospitals was a disgrace to the British nation. A resolution of protest was passed against the action of the Asylums Board, and copies of the protest are to be sent to local members of Parliament.

### A READJUSTMENT OF SALARY.

There are many occasions when increased duties are placed on the medical staff of an institution, but there is not always the same corresponding increase in the amount of remuneration. Some months ago, the Lambeth Board of Guardians, acting on the orders of the Local Government Board, transferred all the children in their workhouses to their schools at Norwood, when they decided that part of the children's infirmary should be used as a nursery. Dr. A. N. V. Johnson, the medical officer, was placed in charge of the new addition, and her salary increased by £25 a year for the extra services. Dr. Smallwood was also attending the old people who were living in the Home for the Aged Poor, but part of this was taken for the accommodation of the children, and consequently the number of people under his care was reduced. The committee have come to the conclusion that there are no powers enabling them to reduce Dr. Smallwood's salary, so that at present they are paying these two medical officers £345 a year. The Guardians have discussed the question of the appointment of a whole-time resident medical officer, who shall have charge of all those in the school and the Home for the Aged Poor, but have decided that the present was not an opportune time to make any alterations.

### THE LEAGUE OF MERCY AND THE WAR.

In order that ladies and gentlemen who reside outside the area in which the League of Mercy has hitherto worked may have an opportunity of assisting the hospitals during the war through the medium of the League, the Grand President, Prince Alexander of Teck, has authorised the formation of a new branch to include the whole of Great Britain outside London and the home counties. The Council of the League has therefore arranged to utilise the machinery of the League for the collection and distribution of funds in aid of the hospital treatment of sick and wounded sailors and soldiers. All who are desirous of obtaining information as to the work of the League or of becoming members are requested to send their names and addresses to the Honorary Secretaries, The League of Mercy, 29 Southampton Street, Strand, W.C.

### "THE INSTITUTIONAL WORKER."

This section of The Hospital contains this week the first of a series of articles dealing with the work, not this time of the different departments, but of the different officials. The subject taken in the first article is "The Hospital Secretary," and as each article is written by a responsible official occupying the post, the duties of which are described, each contribution possesses a personal flavour as well as expert information on its subject. It will be clear that the duties of a hospital secretary vary with the size, nature, and locality of his institution, and there is therefore ample scope for discussion and criticism upon the views put forward and the scope of work defined in the article. Notice is given to the important subject of complaints, and also to new duties imposed upon the secretary by the reception of military patients, and we believe that the articles will be not merely valuable in themselves, but provocative of discussion, questions, and a valuable exchange of views.

### THIS WEEK'S DRUG MARKET.

The market has again been busy. In the case of some drugs, as, for instance, morphine and iodine, the demand is very great indeed, and manufacturers are working under great pressure. The upward movement of prices is still pronounced, and among the articles affected are salicylic acid, aspirin, salicylate of soda, antifebrin, atropine, croton chloral hydrate, cocaine, codeine, phenacetin, and phenazone. Atropine is in very short supply, owing to the heavy demand from the Medical Services of our own and our Allies' Armies; still higher prices will no doubt be reached. Digitalis leaves are dearer. Castor oil has again advanced in price. Permanganate of potash is beginning to advance in price again. The upward movement in the price of cod-liver oil continues in spite of the good results of the Norwegian fishings, the same causes as those mentioned last week continuing to operate. One of the few articles that are cheaper is liquid paraffin, a good medicinal quality of which is now being produced in this country. Liquid extract of male fern is dearer. Stocks of rhubarb are very much reduced. Extract of malt is dearer.

